# Supplementary material for: Splice-Junction-Based Mapping of Alternative Isoforms in the Human Proteome
Source: Cell Rep. Author manuscript; Available in PMC 2020 Jan 15. (PMC6961840; doi:10.1016/j.celrep.2019.11.026)
Supplement: 2 [file NIHMS1546469-supplement-2.zip › DF1/PXD010154/Pancreas/Pancreas_4_MYCBP2_SLLVPVDNIFNASQGVR.pdf]

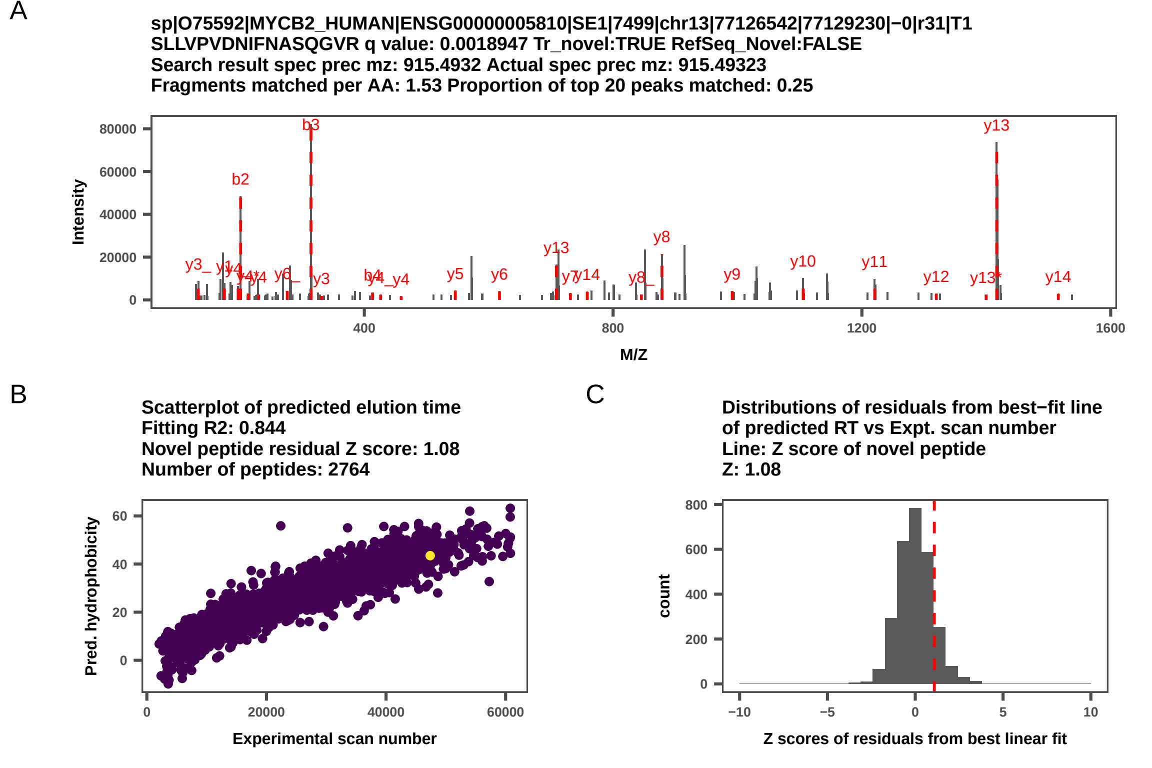
A
sp|O75592|MYCB2_HUMAN|ENSG00000005810|SE1|7499|chr13|77126542|77129230|−0|r31|T1
SLLVPVDNIFNASQGVR q value: 0.0018947 Tr_novel:TRUE RefSeq_Novel:FALSE
Search result spec prec mz: 915.4932 Actual spec prec mz: 915.49323
Fragments matched per AA: 1.53 Proportion of top 20 peaks matched: 0.25
Intensity
80000
60000
40000
20000
0
b2
b3
y13
y8
y5
y6
y9
y10
y11
y12
y14
400
800
1200
1600
M/Z
B
Scatterplot of predicted elution time
Fitting R2: 0.844
Novel peptide residual Z score: 1.08
Number of peptides: 2764
Pred. hydrophobicity
60
40
20
0
0
20000
40000
60000
Experimental scan number
C
Distributions of residuals from best−fit line
of predicted RT vs Expt. scan number
Line: Z score of novel peptide
Z: 1.08
count
800
600
400
200
0
−10
−5
0
5
10
Z scores of residuals from best linear fit
